# Dual targeting of HER3 and MEK may overcome HER3-dependent drug-resistance of colon cancers

**DOI:** 10.18632/oncotarget.11400

**Published:** 2016-08-19

**Authors:** Giulia Bon, Rossella Loria, Carla Azzurra Amoreo, Alessandra Verdina, Isabella Sperduti, Arianna Mastrofrancesco, Silvia Soddu, Maria Grazia Diodoro, Marcella Mottolese, Matilde Todaro, Giorgio Stassi, Michele Milella, Ruggero De Maria, Rita Falcioni

**Affiliations:** ^1^ Department of Research, Advanced Diagnostic, and Technological Innovation, IRCCS Regina Elena National Cancer Institute, Rome, Italy; ^2^ Physiopathology Laboratory of Skin, IRCCS San Gallicano Dermatological Institute, Rome, Italy; ^3^ Surgical and Oncological Sciences, University of Palermo, Palermo, Italy; ^4^ Department of Experimental Clinical Oncology, IRCCS Regina Elena National Cancer Institute, Rome, Italy; ^5^ General Pathology, Catholic University of Rome, Rome, Italy

**Keywords:** colon cancers, HER3, PI3K, MAPK, drug resistance

## Abstract

Although the medical treatment of colorectal cancer has evolved greatly in the last years, a significant portion of early-stage patients develops recurrence after therapies. The current clinical trials are directed to evaluate new drug combinations and treatment schedules.

By the use of patient-derived or established colon cancer cell lines, we found that the tyrosine kinase receptor HER3 is involved in the mechanisms of resistance to therapies. In agreement, the immunohistochemical analysis of total and phospho-HER3 expression in 185 colorectal cancer specimens revealed a significant correlation with lower disease-free survival.

Targeting HER3 by the use of the monoclonal antibody patritumab we found induction of growth arrest in all cell lines. Despite the high efficiency of patritumab in abrogating the HER3-dependent activation of PI3K pathway, the HER2 and EGFR-dependent MAPK pathway is activated as a compensatory mechanism. Interestingly, we found that the MEK-inhibitor trametinib inhibits, as expected, the MAPK pathway but induces the HER3-dependent activation of PI3K pathway. The combined treatment results in the abrogation of both PI3K and MAPK pathways and in a significant reduction of cell proliferation and survival.

These data suggest a new strategy of therapy for HER3-overexpressing colon cancers.

## INTRODUCTION

Colorectal cancer (CRC) is the third most frequent cancer in males and the second in females. The majority of CRC patients have sporadic disease, while genetic mutations are estimated to account for only 5% to 6% of CRC cases overall [1].

The current treatment for CRC is based on combination therapies that in most cases include surgery, local radiotherapy and chemotherapy. 5-FluoroUracil (5-FU) combined with leucovorin and irinotecan (FOLFIRI) is the recommended first-line chemotherapy for CRC; Oxaliplatin (Ox) is used in combination with leucovorin and 5-FU in the FOLFOX regimen. Nevertheless, the development of drug resistance still occurs in a great number of patients determining recurrence.

Several current clinical trials are directed to evaluate new drug combinations, in order to enhance tumor regression, increase overall survival, and improve the quality of life for CRC patients.

In this work, we provide evidence of the crucial role played by the tyrosine kinase receptor HER3 in the development of resistance to 5-FU and Ox treatments in CRC cells. HER3 belongs to the ErbB family of tyrosine kinase receptors, and its up-regulation as well as its activation by over-expression of its ligand, Heregulin-β1 (HRG-β1), are commonly seen in various malignancies [2–5]. In human breast cancers, HER3 overexpression has been reported in 50–70% of cases [6–8] and seems to be associated with metastasis [8], tumor size, and risk of local recurrence [9]. In addition, HER3 protein overexpression has been reported to correlate with resistance to tamoxifen in hormone-dependent breast cancer [10], and resistance to trastuzumab in HER2-overexpressing breast cancer [11]. Increased HER3 mRNA or protein is commonly seen in colon carcinomas and is associated with lymph node metastasis and a shorter time to progression [12–15].

Owing to structural features that limit its intrinsic kinase activity, HER3 cannot be auto-phosphorylated but can be trans-phosphorylated through hetero-dimerization with other family members, such as HER2 [16–18]. Heterodimers of HER3 with other HER family members signal through the PI3K/AKT pathway, which have been implicated in the insurgence of chemoresistance of breast, ovarian, colon, gastric, and lung cancer cells. In particular, the HER-2/HER-3 heterodimer is considered an oncogenic unit, being the strongest activator in nature of the PI3K/AKT pathway [19].

HER3 is a promising onco-therapeutic target [20]. Amongst the many HER3 inhibitors developed and currently in early clinical development [21], patritumab is the first fully humanized monoclonal antibody targeting HER3. Patritumab has shown promising anti-tumor effects in CRC [22] and breast cancer in-vitro and in-vivo cancer models, where it effectively blocked HER3 phosphorylation, degraded HER3, and decreased tumor burden [23–24]. A phase-I clinical study has deemed patritumab safe for patient use and is now being expanded to a phase-II study for treatment of various solid tumor types [25].

Tumor heterogeneity influencing signaling cascades in human cancer affects tumor progression and metastatization [26–27]. The aberrant activation of RAS/BRAF pathway commonly occurs through gain-of-function mutations in genes encoding family members, suggesting that this axis is the regulatory hotspot of the pathway [28–31].

The frequency of mutations and the upstream signals that activate this pathway render ERK molecules good targets for new drugs, with the aim to overcome the mechanism of drug resistance.

Despite the great number of highly specific and highly potent MEK1/2 inhibitors developed and evaluated in clinical studies, only the MEK inhibitor trametinib has gained FDA approval for clinical use, specifically for the treatment of BRAFV600E/K-mutant melanoma [32–33]. Most of the others agents exhibited only limited efficacy as single-agent therapies and failed to demonstrate substantial clinical activity in most tumor types in which they have been studied. Such a lack of response to inhibition of a pathway that is activated in cancer results from activation of compensatory pathways that support cancer cell viability in the presence of the inhibitory drug [34]. Specifically, HER3 has been involved in intrinsic resistance to MEK inhibition [35–36].

These observations, together with our findings supporting the role played by HER3 in the development of resistance to hormone and herceptin therapies [10–11], prompted us to test a new drug combination, involving the monoclonal antibody patritumab, targeting HER3, and the MEK-inhibitor trametinib. Our results suggest that this combination protocol could represent a new therapeutic approach to escape drug-resistance in HER3-overexpressing CRC.

## RESULTS

### HER3 is involved in the mechanisms of resistance of colon cancer cells to chemotherapy

In order to evaluate the involvement of HER3 in the responsiveness of colon cancer cells to chemotherapies, we analyzed three patient-derived colon cancer cell lines (Pt-1, Pt-2, Pt-3) and the established colon cancer cell line HT29. All cell lines were characterized for the mutations of BRAF, KRAS, and PIK3CA oncogenes, for the status of p53 (Supplementary Table S1), and for the expression of EGFR family members, whose expression and activity has been described strictly correlated with the responsiveness to therapy (Supplementary Figure S1) [10–11, 37].

**Figure 1:**
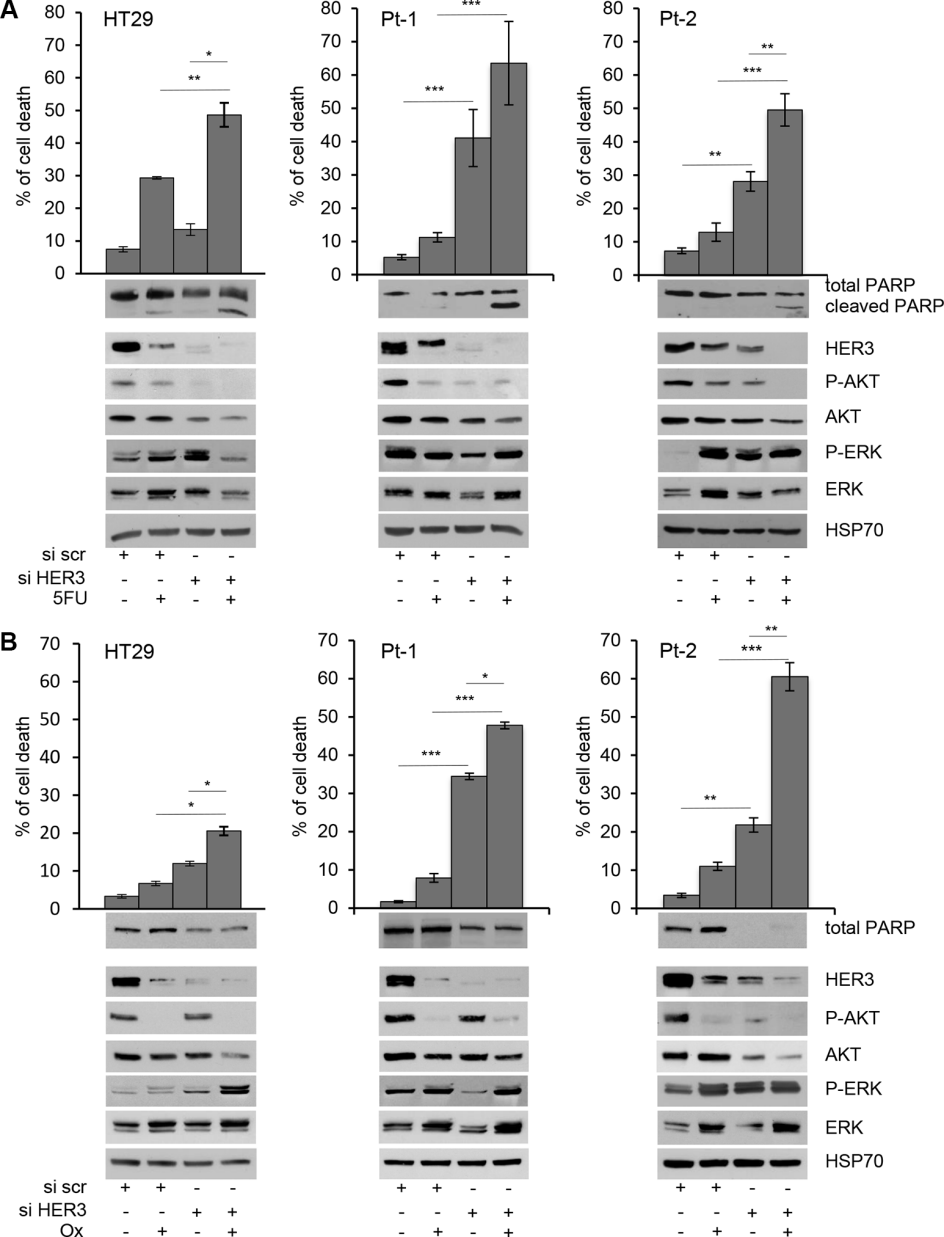
Depletion of HER3 expression induces apoptosis and sensitizes colon cancer cells to 5-FluoroUracil and Oxaliplatin Cell viability of transiently transfected siscr and siHER3 HT29, Pt-1, and Pt-2 cells, treated or not with 5-FU (**A**) upper panel) and Ox (**B**) upper panel) for 48 hours, was evaluated by Trypan Blue exclusion. The percentage of cell death is presented as mean +/– standard deviation of three independent experiments. Total cell lysates from transiently transfected siscr and siHER3 HT29, Pt-1, and Pt-2 cells, treated or not with 5-FU (A, lower panel) and Ox (B, lower panel) were analyzed by immunoblot to evaluate the expression of HER3, total and p-AKT, total and p-ERK, and PARP. The anti-HSP70 antibody was used to validate equivalent amount of loaded proteins in each lane. The results are presented as mean +/– standard deviation of three independent experiments ^*^p < 0.05, ^**^p < 0.001, ^***^p < 0.0001.

We found that the depletion of HER3 by specific siRNA (siHER3) induces per se cell death in Pt-1 and Pt-2 patient-derived cell lines, whereas it has no significant effect in HT29 colon cancer cells (Figure 1A and 1B, upper panels). Interestingly, siHER3 rescues the responsiveness of Pt-1 and Pt-2 cells to 5-FU and Ox treatments (*p* < 0.05 and *p* < 0.001), which are almost ineffective per se. Even though the *in vitro* treatment with 5-FU and Ox alone induces down-regulation of HER3, the residual expression of the receptor is sufficient to induce drug-resistance (Figure 1A and 1B, upper panels). HT29 cells showed a partial response only to 5-FU, but not to Ox (Figure 1A and 1B, upper panels); nevertheless the depletion of HER3 in combination with chemotherapy in HT29 is not as effective as in patient-derived cell lines. The combined treatment induces significant levels of apoptosis, as indicate by PARP cleavage (Figure 1A, lower panels), or by reduction of total PARP (Figure 1B, lower panels). These results were further validated by TUNEL assay showing that siHER3 alone and in combination with 5FU or Ox induces cell death by apoptosis (Supplementary Figure S2A and S2B). These data strongly suggest that HER3 and the signaling pathways downstream could be involved in the mechanisms of resistance to chemotherapy in colon cancer cells. In agreement with this hypothesis, we found a strong down-regulation of PI3K/AKT signaling pathway in HER3-depleted cells upon 5-FU or Ox treatment, as indicated by the inhibition of AKT phosphorylation (Figure 1A and 1B, lower panels). The above results were confirmed by the use of a second specific siRNA to interference with HER3 expression (Supplementary Figure S3).

Chemotherapy-induced genotoxic stress often activates multiple signal transduction pathways such as MAPK, aberrantly activated in CRC patients [38], which has been associated with anti-apoptotic processes and chemo-resistance development [39]. It has been hypothesized that MAPK activation may provide cancer cells with survival advantage and assist malignant cells escaping from the drug-induced apoptotic challenge [40]. In such a perspective, we also evaluated the phosphorylation levels of MAPK and found that both 5-FU and Ox treatments, alone or in combination with HER3 depletion induce the up-regulation of p-ERK in HT29 and in Pt-2 cells while the results it is not evident in Pt-1 cells (Figure 1A and 1B, lower panels, and Supplementary Figure S4). These results are in agreement with recent findings indicating that one of the mechanisms of resistance to therapies involves the activation of compensatory pathways to which tumor cells become addicted [41–42].

### Total and phospho-HER3 expression correlate with disease-free survival

To validate our *in vitro* data we analyzed, by immunohistochemistry (IHC), the expression level of total- and phospho-HER3 in specimens derived from patients surgically treated at the Regina Elena National Cancer Institute between 2000 and 2013. We correlated the expression of both isoforms with the Disease-Free Survival (DFS). To this end, we randomly selected 185 CRC patients with a median follow-up of 66 months (95% CI 61.8–71.5), including 135 colon and 50 rectal carcinomas that were retrospectively evaluated for DFS.

As summarized in Table 1, 21 patients (11%) were T1-2, 122 (66%) T3 and 42 (23%) T4. Eighty-six (46%) patients were lymph node negative and 99 (54%) were node positive. Furthermore, 153 (83%) CRC were graded, using the Bloom and Richardson scoring system, as well/moderate differentiated (G1-G2), and 32 (17%) as poorly differentiated (G3). Pathological staging identified 73 (39%) and 68 (37%) tumors at stages I–II and III respectively and 44 (24%) at stage IV. Tumors were staged according to the Unione Internationale Contre le Cancer tumor-node-metastasis system criteria (TNM Ed.7, 2009). The study was reviewed and approved by the ethics committee of the Regina Elena National Cancer Institute.

**Table 1:** Bio-pathological characteristics of 185 colorectal carcinomas

Characteristics	N°	%
**Number of patients**	**185**	
**Tumor size**		
T1-2	21	11
T3	122	66
T4	42	23
**Lymph node status**		
Negative	86	46
Positive	99	54
**Grading**		
G1-2	153	83
G3	32	17
**Stage**		
I–II	73	39
III	68	37
IV	44	24
**HER3 status**		
0	17	9
1+	56	30
2+	98	53
3+	14	8
**pHER3 status**		
0	80	43
1+	2	1
2+	85	46
3+	18	10

185 CRC cases were classified for tumor size, lymph node status, grading, and stage, total and phospho-HER3 status.

**Figure 2:**
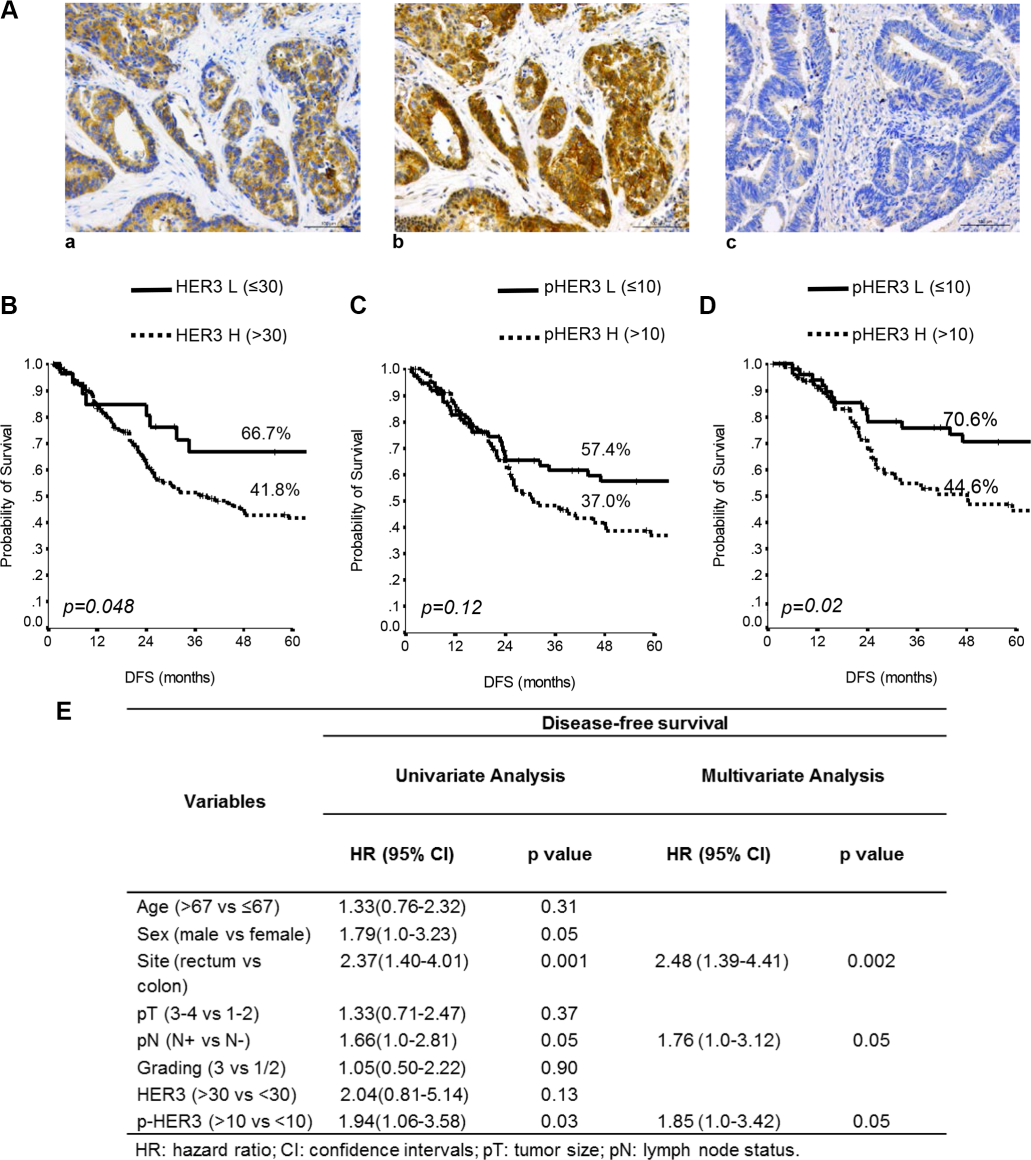
Total and p-HER3 expression correlates with disease-free survival in CRC specimens (**A**) Representative CRC cases showing: (a) total HER3 positivity, (b) phospho-HER3 positivity on the consequential section, (c) negative HER3 expression. Scale bar 100 µm. Kaplan-Meier estimates DFS for HER3 (**B**) and pHER3 status (**C**) in 185 stage I–IV CRC patients. (**D**) Estimates of DFS for pHER3 status in the I–III stage CRC patients. *P*-values were calculated using the long-rank test. DFS = Disease-free Survival. L = Low expression, H = High expression. (**E**) Univariate and multivariate analyses performed in the 141 stage I–III patients.

One hundred and sixty eight (91%) and 105 (57%) CRC patients were found positive for HER3 and pHER3, respectively (Table 1, Figure 2A a, b). Figure 2A c shows the IHC analysis of a representative HER3 negative specimen. In Supplementary Figure S5 are reported the levels of total- and pHER3 expression scored semi-quantitatively based on staining intensity (0, 1+, 2+, and 3+) whose percent of distribution, using the IRS described in statistical analysis section, is reported in Figure 2B–2D.

When the entire series of 185 cases with complete follow-up data was analyzed by unadjusted Kaplan-Meier curves, low HER3 was able to identify subgroups of patients at lower risk of relapse (*P* = 0.048) (Figure 2B). By contrast, in the entire series of 185 patients stage I–IV, pHER3 expression had no discernible effect on DFS (*P* = 0.12) (Figure 2C). However, when we excluded from this analysis the patients at stage IV, which is linked to many additional modifications that induce a profound change in the biology of tumors, in the remaining 141 patients, stage I–III, high pHER3 expression predicted significantly shorter DFS (*p* = 0.02) (Figure 2D).

Univariate and multivariate analyses (Cox model) further confirmed these data, identifying high expression (score > 10) of pHER3 as a significant predictor of worse DFS in the 141 patients described above (HR 1.94, CI 1.06 to 3.58, *p* = 0.03 and HR 1.85, CI 1.0 to 3.42, *P* = 0.05) (Figure 2E). Interestingly, we found that in a subset of lower pHER3 patients (stage I-IV), the higher pHER3 expression in patients with stage I-III predicted a significant shorter DFS (*p* = 0.0009) (Supplementary Figure S6A, and S6B).

### Inhibition of HER3 activity by patritumab induces growth arrest in patient-derived colon cancer cells Pt-1 and Pt-2

**Figure 3:**
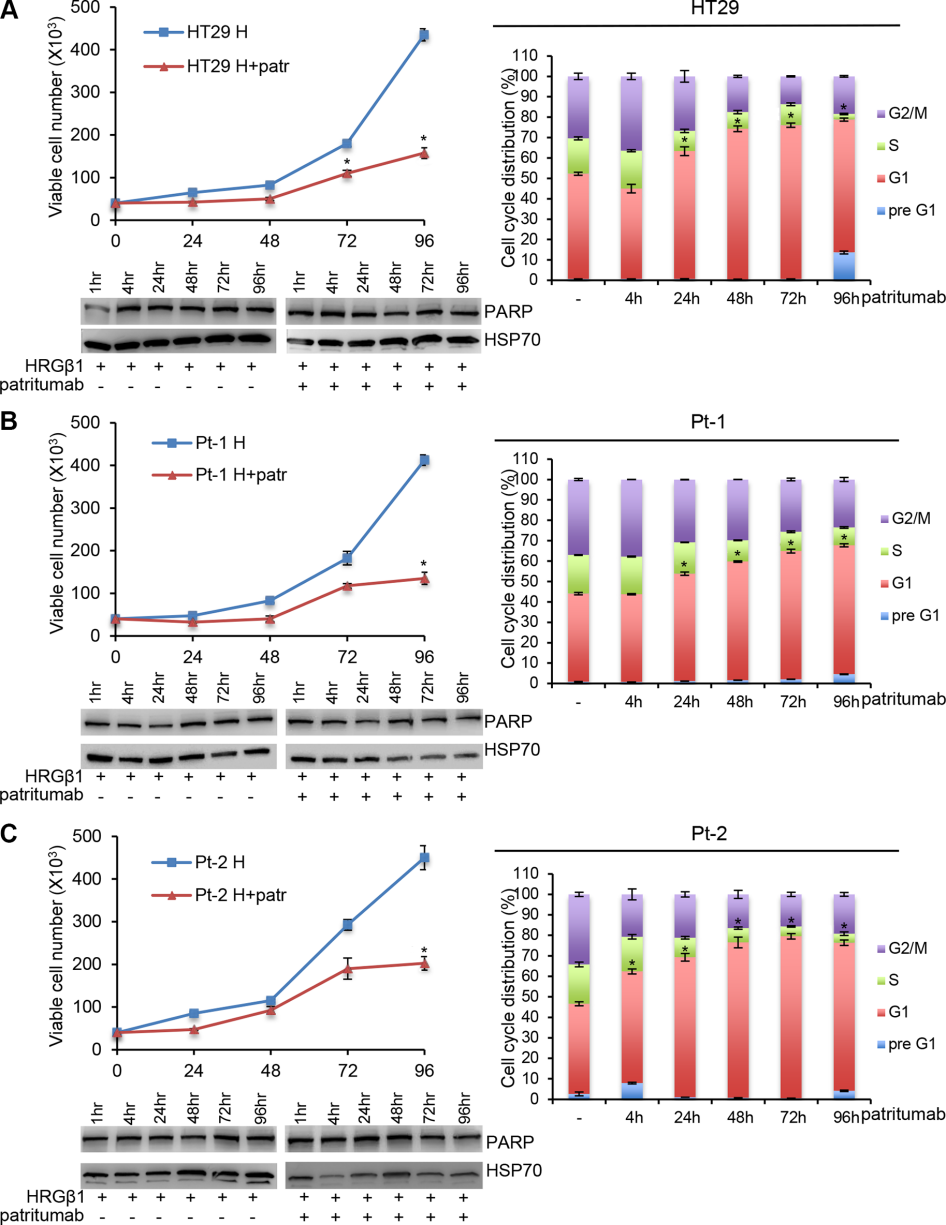
Patritumab administration inhibits cell proliferation and induces growth arrest in the G1 phase of the cell cycle 5 × 10^4^ cells of HT29 (**A**), Pt-1 (**B**), and Pt-2 (**C**) cell lines were seeded into six-well plates. HRG-β1-stimulated cells, patritumab-treated or untreated, were collected at the indicated times and counted in 0.2% trypan blue solution (left, upper panels). The cells incubated with 10 ng/ml HRG-β1 and 10 μg/ml patritumab antibody for the indicated times were lysed and analyzed by immunoblot to evaluate the expression of PARP. Anti-HSP70 antibody was used to validate equivalent amount of loaded proteins in each lane (left, lower panels). The same cells were stained in PBS containing propidium iodide and RNase-A overnight at 4°C. The DNA content of the cells was measured by fluorescence-activated cell sorting (FACS) (right panels). A total number of 1 × 10^4^ cells for each sample were acquired. The results are presented as mean +/– standard deviation. ^*^p < 0.05; ^**^p < 0.001; ^***^p < 0.0001 compared to control.

Patritumab is the first fully human monoclonal immunoglobulin G1 (IgG1) antibody directed against HER3 binds specifically to the extra-cellular domain of HER3 [22]. In order to verify the growth inhibition ability previously showed by this antibody in multiple tumor cell lines, we analyzed its effect in patient-derived colon cancer cell lines. In order to render the patritumab efficient in our cell lines, that do not express a constitutively active form of HER3 tyrosine kinase receptor, we treated all the cell lines with HRG-β1 to induce HER3 phosphorylation, making the receptor targetable for the antibody. We found that the treatment of HT29, Pt-1 and Pt-2 cell lines with patritumab, under stimulation with HRG-β1, induces a significant inhibition of cell proliferation (Figure 3A, 3B and 3C, upper left panels). A more detailed analysis confirmed that the treatment with patritumab does not induce apoptosis; indeed the analysis of total cell lysates from samples collected after 4, 24, 48, 72 and 96 hours of incubation with patritumab, revealed that the total levels of PARP remain unaffected (Figure 3A, 3B and 3C, left lower panels). We then checked for the cell cycle distribution of cells collected at the time points of the same experiment and we found that patritumab treatment inhibits cell cycle progression inducing a block of the cells at the G1 phase of the cell cycle. The accumulation of patritumab-treated cells at the G1 phase reached the maximum level between 72 and 96 hours post-treatment in the cell lines analyzed (Figure 3A, 3B and 3C, right panels). These data allow us to confirm that patritumab induces growth arrest also in patient-derived colon cancer cells.

### Patritumab inhibits PI3K survival pathway and induces the phosphorylation of ERK as a compensatory mechanism

**Figure 4:**
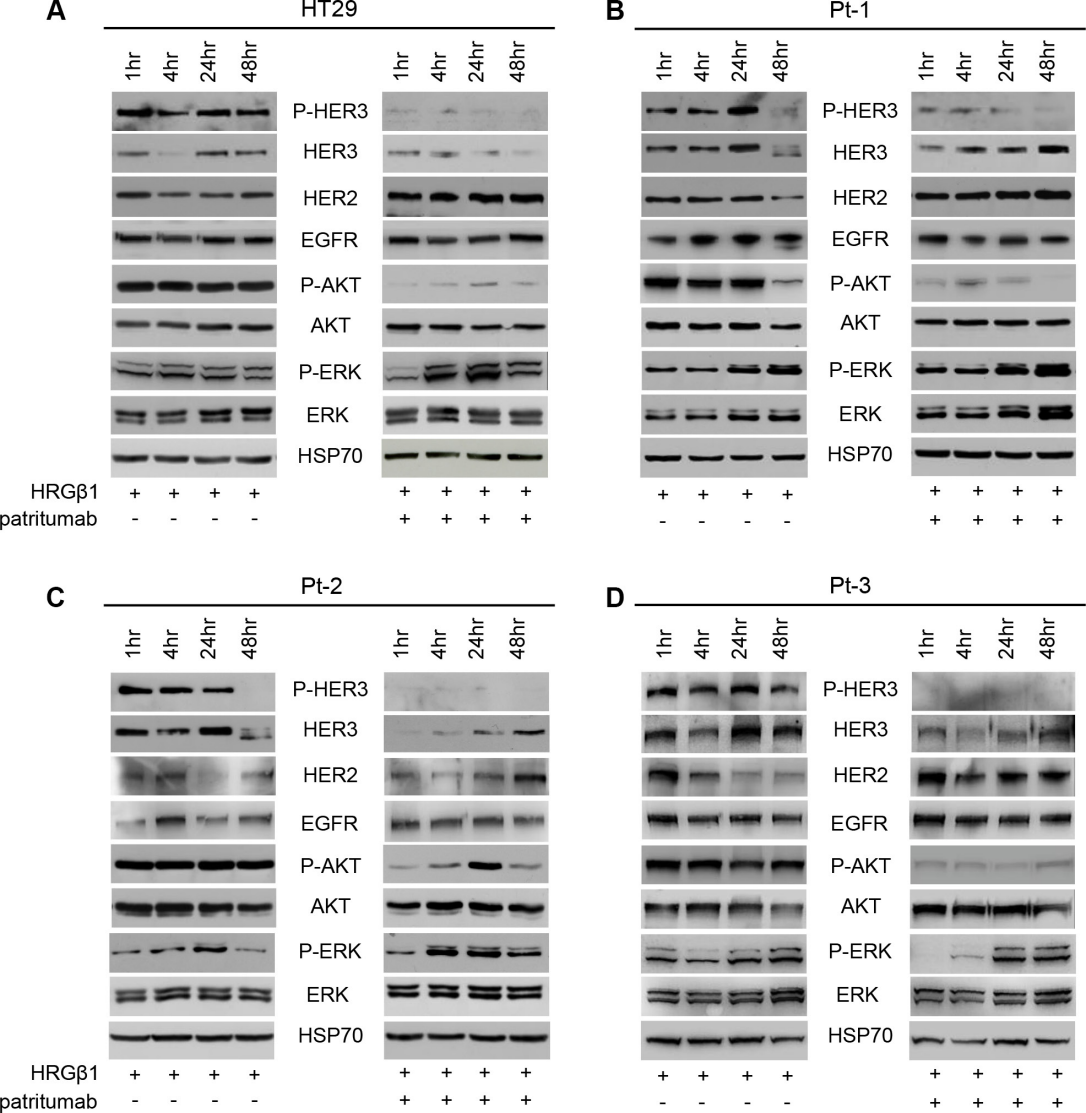
Patritumab administration inhibits the PI3K survival pathway and activates the MAPK proliferation pathway Total cell lysates from HT29 (**A**), Pt-1 (**B**), Pt-2 (**C**), and Pt-3 (**D**) cells, incubated with 10 ng/ml HRG-β1 and 10 μg/ml patritumab antibody for the indicated times, were analyzed by immunoblot to evaluate the expression of total and P-HER3, HER2, EGFR, total and p-AKT, total and p-ERK. The anti-HSP70 antibody was used to validate equivalent amount of loaded proteins in each lane.

It has been shown that patritumab treatment induces rapid internalization and degradation of HER3 receptor [22]. Further evidence showed that this antibody inhibits the PI3K survival pathway in lung cancer and head and neck cell lines [43]. To analyze the molecular perturbations induced by patritumab treatment in the patient-derived colon cancer cell lines Pt-1, Pt-2, Pt-3 and in the colon cancer cell line HT29, we incubated all cell lines with patritumab under stimulation with HRG-β1. The stimulation induces a rapid activation of HER3 and AKT, measured as phosphorylation levels, in all cell lines up to 24 h of treatment (Figure 4A–4D, left panels). By contrast, patritumab treatment abrogates the HRG-β1-induced HER3 phosphorylation and, as expected, induces rapid internalization and degradation of the receptor that results in the abrogation of AKT phosphorylation (Figure 4A–4D, right panels) compared to control cells (Figure 4A–4D, left panels).

Interestingly, we also found that patritumab treatment results in a significant up-regulation of total HER2 in all cell lines analyzed (Figure 4A–4D right panels *vs* left panels). This data was further confirmed by FACS analysis (Supplementary Figure S7A). By contrast, patritumab treatment does not modulate significantly EGFR in HT29 and in Pt-1 cells, while we observed a significant increase of the receptor in Pt-2 and Pt-3 cells (Figure 4C and 4D). This result is in agreement with recent observations indicating that cancer cells respond to targeted inhibitors by adapting their signaling circuitry, often in terms of activation of compensatory signaling mechanisms [42]. Indeed, the increase of HER2 and EGFR receptors induced by patritumab treatment resulted in the activation of MAPK pathway in all treated cells compared to control cells (Figure 4A–4D, right *vs* left panels). Densitometry analysis of HER2 and EGFR are reported in Supplementary Figure S7B. The involvement of both receptors in the compensatory mechanism promoted by patritumab treatment was confirmed by blocking the signaling of EGFR and HER2 by erlotinib and trastuzumab, respectively. Indeed, in the presence of patritumab, the inhibition of both receptors does not allow the activation of MAPK (Supplementary Figure S8).

### The MEK-inhibitor trametinib activates the HER3-dependent PI3K survival pathway

**Figure 5:**
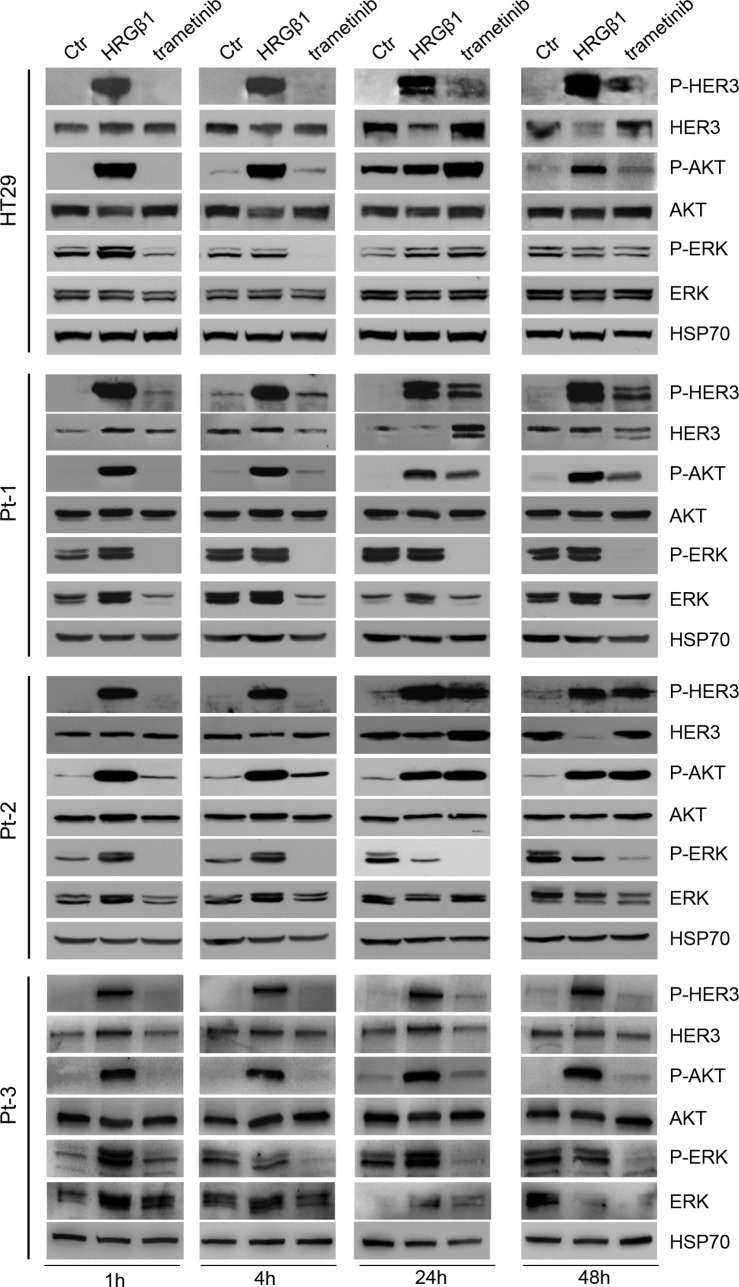
Trametinib administration induces the HER3-dependent activation of PI3K pathway Total cell lysates from HT29, Pt-1, Pt-2, and Pt-3 cells incubated with 10 ng/ml HRG-β1 or treated with 5 nM trametinib, for the indicated times, were analyzed by immunoblot to evaluate the expression of total and p-HER3, total and p-AKT, total and p-ERK. The anti-HSP70 antibody was used to validate equivalent amount of loaded proteins in each lane.

Since the treatment of colon cancer cell lines with patritumab down-regulates PI3K signaling but results in the activation of MAPK pathway as a compensatory mechanism, we decided to test the effect induced by the MEK-inhibitor trametinib. It has been previously described that the MEK inhibitor trametinib rapidly induces hyper-phosphorylation of HER3 tyrosine kinase receptor in a panel of melanoma cell lines harboring a variety of BRAFV600E/K mutations [35]. The authors showed that up-regulation of phospho-HER3 is due to an autocrine loop involving increased transcription and production of neuregulin by melanoma cells. HER3 has been involved in intrinsic resistance to MEK inhibition also in KRAS mutant lung and colon cancers [36] and it has been found that MEK inhibition by selumatenib results in MYC-dependent transcriptional up-regulation of HER3, which is responsible for intrinsic drug resistance. Thus, we treated our Pt-1, Pt-2, Pt-3, and HT29 cells with trametinib, in order to evaluate the responsiveness of the cells to the drug. As expected, trametinib treatment induced a rapid inhibition of ERK phosphorylation in all cell lines, which was evident 1 hour following exposure to the drug and was maintained up to 48 hours (Figure 5). Interestingly, we also found that trametinib treatment determines a rapid and strong induction of HER3 phosphorylation, resulting in AKT activation, between 24 and 48 hours of treatment in all cell lines (Figure 5). The densitometry analysis of p-Akt and p-MAPK reported in Supplementary Figure S9 confirms the above results. This last result is in agreement with previous data showing that the MEK inhibitor semulatenib induces HER3 transcriptional up-regulation and phosphorylation *in vivo* in KRAS mutant lung and colon cancers [36].

Our data confirm that MEK inhibition by trametinib induces the HER3-dependent activation of PI3K survival pathway independently of the status of BRAF and KRAS oncogenes, and suggest that the compensatory mechanism activated by the MEK inhibitor could be independent from the genetic background of the cells.

### Dual targeting of HER3 and MEK impairs colon cancer cell viability

**Figure 6:**
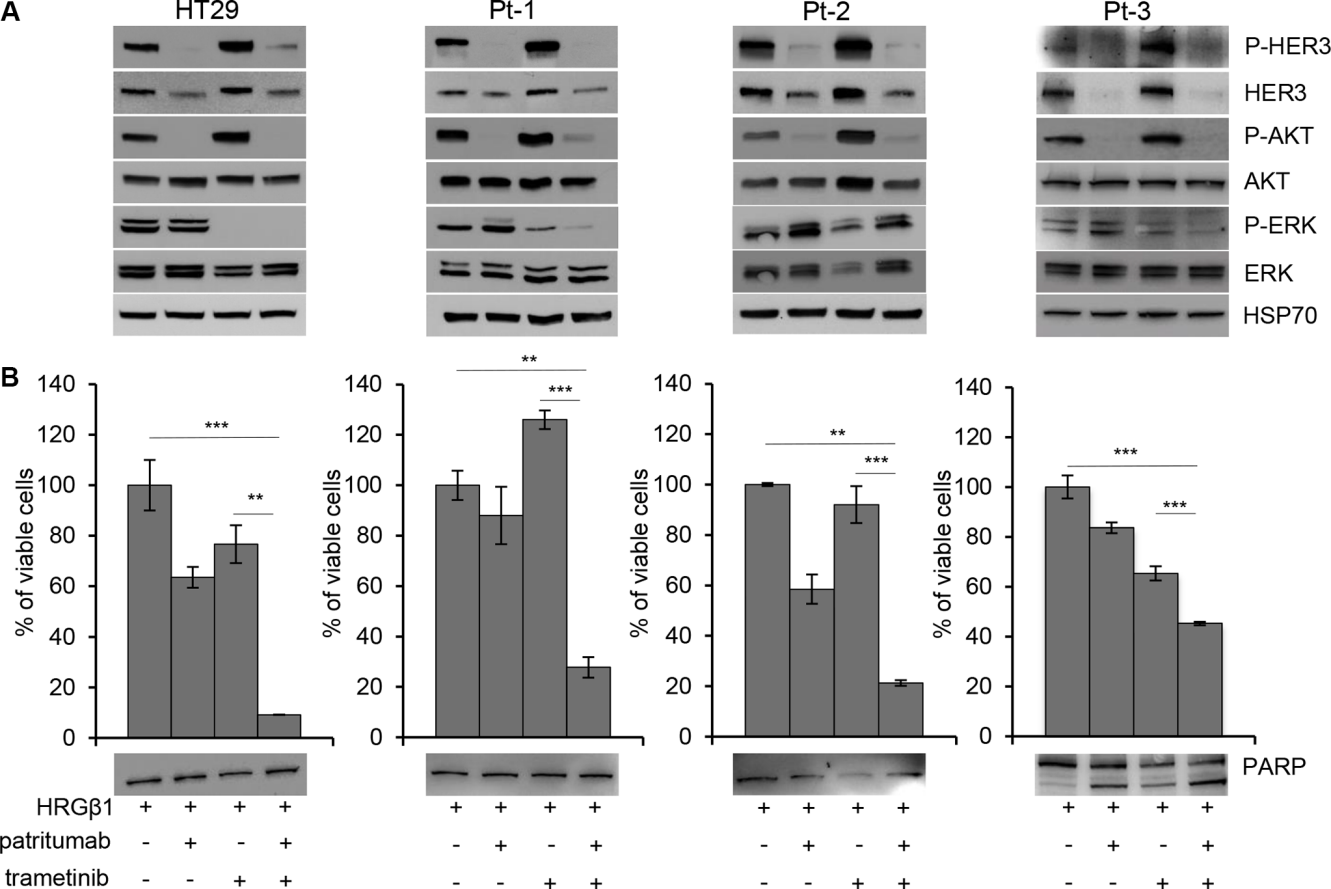
Dual targeting of HER3 and MEK impairs viability of colon cancer cells (**A**) Total cell lysates from HT29, Pt-1, Pt-2, and Pt-3 cells incubated with 10 ng/ml HRG-β1, 5 nM trametinib, 10 μg/ml patritumab antibody, or a combination of trametinib and patritumab antibody, for 24 hours, were analyzed by immunoblot to evaluate the expression of total and p-HER3, total and p-AKT, total and p-ERK. The anti-HSP70 antibody was used to validate equivalent amount of loaded proteins in each lane. (**B**) Cell viability of HT29, Pt-1, Pt-2 and Pt-3 cells treated with 10 ng/ml HRG-β1, 5 nM trametinib, 10 μg/ml patritumab antibody, or a combination of trametinib and patritumab antibody for 72 hours, was evaluated by Crystal Violet assay. The results are presented as mean +/– standard deviation of three independent experiments. ^**^p < 0.001; ^***^p < 0.0001 combined treatment compared to control and to trametinib alone.

According to previous data, we targeted both PI3K and MAPK pathways by treating colon cancer cells with patritumab in combination with trametinib. As expected, upon stimulation with HRG-β1, the single treatment with patritumab inhibits PI3K but activates MAPK pathway; on the contrary, trametinib abrogates ERK but activates HER3 receptor (Figure 6A, upper panels). The treatment with both inhibitors resulted in abrogation of both pathways (Figure 6A, upper panels); indeed Pt-1, Pt-2 and Pt-3 patient-derived colon cancer cells treated with the combined therapy show the inhibition of HER3, AKT, and ERK phosphorylation indicating that the compensatory activation of the two pathways induced by the single drug results in a strong response when they are administrated in combination. The dual inhibition by patritumab and trametinib induces cell death by apoptosis in Pt-3 cells, reaching a 40% of cell death upon 72 h (Figure 6B, lower panel). The same treatment does not induce cell death in Pt-1, Pt-2, and HT29, as suggested by PARP cleavage (Figure 6B, lower panels), but the viability of these cells is severely impaired, suggesting that the dual therapy induces a block of cell cycle progression. These results were confirmed by TUNEL assay showing that patritumab and trametinib treatments alone or the combination of both induces apoptosis only in Pt-3 cells (Supplemnary Figure S10A and S10B).

## DISCUSSION

The development of resistance to therapy remains an unresolved issue in the treatment of CRC. The number of proteins and signaling pathways implicated in the mechanisms of drug resistance has increased through the last years, and it has been well established that somatic alterations in key components of these pathways can function as driver mutations during oncogenesis. A number of these cancer drivers, as well as other core proteins in the signaling pathways, have received much attention from the pharmaceutical industry as ‘‘drugable’’ targets for tumor therapy. Moreover, it is clear that tumors can contain a high degree of molecular heterogeneity [44], thus drug resistance can arise through therapy-induced selection of a resistant minor subpopulation of cells present in the tumor. Therefore it is increasingly clear the need to hit multiple target pathways to overcome acquired resistance.

Among various alterations, up-regulated RTK signaling, due to mutation or amplification of the receptors, contributes to a variety of human malignancies, with members of the ErbB family being prominent cancer drivers. In these tumors the RAF/MEK/ERK and PI3K/AKT/mTOR effector pathways are all activated [45].

Our data demonstrate that the expression and activity of HER3 correlate with lower DFS in a cohort of 185 specimens of colorectal cancer. We also show that HER3 is responsible, at least in part, for the acquired resistance of patient-derived CRC cells to 5-FU and Ox drugs. Indeed, the interference with HER3 expression induces cell death and sensitizes cancer cells to chemotherapy. Interestingly, patritumab treatment induces the up-regulation of HER2 and EGFR, which in turn activate MAPK pathway suggesting that a mechanism of signaling redundancy is induced by patritumab. A similar response has been reported in HER2-expressing breast cancer cells. In this case, inhibition of PI3K or AKT has been found to promote the expression of HER3 causing increased HER2/HER3 signaling and compensatory activation of the ERK cascade [46]. Other reports suggest that HER3 signaling and sustained PI3K/AKT activation are induced upon HER2 and EGFR-targeted therapy, and that patritumab can overcome the resistance induced by heregulin [47–49].

The ERK cascade is an important effector pathway that is upregulated in a high percentage of human tumors [50]. ERK signaling is driven by oncogenic RAS and RTK proteins, as well as by mutational activated BRAF proteins. BRAF and KRAS mutations are present in approximately 5–15% and 50% of cases, respectively [51–53]. The V600E BRAF mutation clearly functions as a driver mutation in colorectal cancer. However, unlike BRAF-mutated melanoma, single-agent BRAF inhibition has been an ineffective strategy in BRAF-mutated colorectal cancer.

The differential sensitivity to BRAF inhibition displayed by BRAF-mutated melanoma and colorectal cancers may be partly explained by the EGFR protein. A key observation is that the vast majority of colorectal cancer expresses EGFR and the inhibition of BRAF triggers the activation of EGFR. The combined EGFR and BRAF inhibition decreases cell proliferation and triggers apoptosis of BRAF-mutated colorectal cancer cell lines and xenografts [54]. On the contrary, melanomas do not express EGFR but forced expression of EGFR in melanoma cells leads to resistance to vemurafenib [55]. These preclinical observations led to the current clinical trials combining inhibitors of BRAF and EGFR. On the other hands, both mutations in KRAS and BRAF have been clearly demonstrated to predict resistance to EGFR-directed therapy in colorectal cancer [56–57]. A further difference between BRAF-mutated melanoma and colorectal cancer is that BRAF-mutated colorectal cancer cells possess higher levels of PI3K activation than BRAF-mutated melanoma cells, and that resistance to vemurafenib could be overcome by combining vemurafenib with PI3K inhibitors [58–60].

We show here that the MEK-inhibitor trametinib activates HER3-dependent PI3K pathway independently of the mutation status of KRAS and BRAF. Indeed we observed the same mechanism in three patient-derived colon cancer cell lines carrying BRAFV600E (Pt-1), KRASG13 (Pt-3) and wt BRAF and KRAS (Pt-2). Our results are in agreement with previous study demonstrating that in response to MEK inhibition colon cancer shows up-regulation of RTKs [36, 60]. Furthermore, HER3 up-regulation as a consequence of MEK inhibitor was observed in BRAF mutant thyroid carcinomas and melanomas [61], whereas trametinib was shown to induce hyper-phosphorylation of HER3 in BRAFV600E/K melanoma [35], and selumetinib-induced resistance in KRAS mutant lung and colon cancer is due to the transcriptional induction of HER3 [36]. In agreement, our results demonstrated that trametinib causes a strong suppression of MEK-ERK signaling in CRC, whereas AKT signaling was even increased in the presence of the inhibitor. Dual inhibition of MEK and HER3, obtained by combining trametinib and patritumab, determined the complete inhibition of both ERK and AKT signaling pathways resulting in a severely impaired viability of CRC cells.

Our finding could have important implications, leading us to suggest a specific therapeutic strategy for a subgroup of HER3-overexpressing CRCs.

## MATERIALS AND METHODS

### Cell lines

The colon cancer cell line HT29, carrying mutant BRAFV600E and PIK3CAP449T, was obtained from the American Type Culture Collection (ATCC) and maintained in DMEM medium supplemented with 10% FCS, 1% penicillin/streptomycin and 1% glutamine (Invitrogen, Milan, Italy). The patient-derived colon cancer cell lines: Pt-1 carrying mutant BRAFV600E and PIK3CAP449T; Pt-2 carrying wild type (wt) BRAF and mutant PIK3CAH1047R; Pt-3 carrying mutant KRAS G13 and mutant PIK3CAE542K were kindly provided by Istituto Superiore di Sanità (ISS) and maintained in DMEM medium supplemented with 10% FCS, 1% penicillin/streptomycin and 1% glutamine (Invitrogen, Milan, Italy). All the cell lines, with the exception of Pt-3 cells, carry mutant p53.

### Treatments

To induce the phosphorylation of HER3, the cells were stimulated with HRG-β1 (R&D Systems, Milan, Italy), at the concentration of 10 ng/ml for the indicated times. The anti-HER3 antibody patritumab was kindly provided from Daiichi Sankyo (Tokyo, Japan) was used at the concentration of 10 μg/ml. The chemotherapeutic drugs 5-FU and Ox were purchased from Teva Italia (Assago, Mi) and used at the concentration of 50 and 20 μM, respectively. The anti-MEK inhibitor trametinib (GSK-1120212) (Selleckchem, Milan, Italy) was used at the concentration of 5 nM. The treatment were performed at the indicated times.

### Antibodies

Rabbit anti-phospho-ser473 (#9271) and total-AKT (#9272), anti-phospho- (#9101L) and total-ERK 1/2 (#9102), anti-PARP (#9542), anti-phospho- (#2247) and total-HER2 (#2165), anti-phospho- (#3777) and total-EGFR (#4267), and anti-phospho-HER3 (#4791) antibodies were purchased from Cell Signaling (Milan, IT). Rabbit anti-HER3 (sc-285) antibody was purchased from Santa Cruz Biotechnology (Milan, IT), and mouse anti-HSP70 (N27F34) antibody was purchased from Stressgen (Milan, IT). These antibodies were used in Western Blot experiments. Rabbit anti-HER2 (#554299), purchased from BD Biosciences (Milan, IT), was used in immunofluorescence (FACS) analysis experiments. Total and phospho-HER3 (#12708 and #4791, respectively) were purchased from Cell Signaling and used for immunohistochemistry (IHC).

FITC and Peroxidase-conjugated secondary antibodies anti-IgGs were purchased from Cappel and/or BioRad (Milan, IT).

### Western blot analysis

To analyze phospho and total-HER3, phospho and total-HER2, phospho and total-EGFR, phospho- and Total-ERK 1/2, PARP, and HSP70 proteins expression, the cells were lysed with RIPA buffer [50 mM Tris (pH 8), 150 mM NaCl, 1% Nonidet P40, 0,1% deoxycholate, 0,1% SDS, 1 mM PMSF, 5 mM Na_3_VO_4_, 50 mM protease inhibitors (SIGMA-Aldrich, Milan, IT)] for 30 minutes at 4°C. Total cell lysates were clarified by centrifugation at 14,000 rpm for 30 minutes at 4°C. To analyze P-AKT the cells were washed three times with cold PBS, lysed with NP40 buffer [(1% Nonidet P40, 10% glycerol, 137 mM NaCl, 20 mM Tris HCl (pH 7,4), 50 mM NaF, 1 mM PMSF, 5 mM Na3VO4, 50 mM protease inhibitors (SIGMA-Aldrich, Milan, IT)] and incubated on ice for 20 minutes. Total cell lysates were clarified by centrifugation at 14,000 rpm for 30 minutes 4°C. Aliquots of cell extracts containing equivalent amount of proteins were treated at 65°C for 5 minutes, resolved by SDS-polyacrilamide gradient gel electrophoresis 8–16% (SDS-PAGE), and transferred to nitrocellulose. The horseradish peroxidase-conjugated goat anti-mouse or ant-rabbit were used as secondary antibodies. Signals were detected by LuminataTM Classico Western HRP substrate (Millipore). Same amount of total protein from three independent experiments were pooled and analyzed. Total loaded proteins were normalized by anti-HSP70.

### Flow cytometry analysis

The expression level of HER2 in HT29, Pt-1, and Pt-2 cells was detected by flow cytometry analysis of stained cells. In brief, cells harvested using citrate saline buffer (0.134 M KCl, 0.015 M Na citrate) were washed twice with cold PBS containing 0.002% EDTA and 10 mM NaN_3_ (washing buffer). Samples of 1 × 10^6^ cells, treated as indicated, were incubated for 1 h at 4°C with saturating concentrations of anti-HER2 diluted in PBS containing 0.5% bovine serum albumin (BSA). The cells were then washed three times with washing buffer (PBS containing 0.5% BSA) and incubated for 1 h at 4°C with 50 µl of FITC-conjugated secondary antibodies [F(ab')2 diluted 1:20 in PBS/BSA]. After three washes, the cells were suspended in 1 ml of washing buffer. Cell suspensions were analyzed on a FACSCalibur flow cytometer (Becton-Dickinson, San Josè, CA, USA) equipped with a 488 nm argon laser. A total number of 1 × 10^4^ cells for each sample was acquired. The Cell-Quest software was used to analyze data.

### RNA interference

To inactivate HER3 expression (siHER3), cells were transiently transfected with Lipofectamine 2000 (Invitrogen, Milan, Italy) following the manufacturer procedures with either two different HER3 anti-sense double strand siRNA:

1) 5′-GCUCUACGAGAGGUGUGAGTT-3′ 5′-CUCACACCUCUCGUAGAGCTT-3′

2) 5′-GAGCGACUAGACAUCAAGCTT-3′ 5′-GCUUGAUGUCUAGUCGCUCTT-3′ or a scrambled anti-sense double strand siRNA (siscr)

5′-GCGCGCAACUCUACCUCUATT-3′ 5′-UAGAGGUAGAGUUGCGCGCTT-3′.

The oligonucleotides were synthesized by Oligoengine Inc. (Seattle, WA).

### Cell proliferation and cell death

HT29, Pt-1, and Pt-2 cells (5 × 10^4^) were plated onto six-well plates. After an overnight of serum starvation, the cells were incubated with 10 ng/ml HRG-β1 (ctr cells) and 10 μg/ml patritumab antibody for the indicated times. Every 24 h cells were collected and counted in 0.2% trypan blue solution, whereas medium changes were performed for the remaining samples. Each assay was repeated at least three times.

HT29, Pt-1, Pt-2, and Pt-3 cells (5 × 10^5^) were plated onto 60 mm dishes. The day after, the cells were transiently transfected with si-scr or si-HER3. After 24 h the cells were treated with 50 mM 5-FU or 20 mM Ox alone or in combination with si-HER3 for 48 hrs. The viability of the cells was evaluated by trypan blue exclusion or by TUNEL assay. For the treatment with patritumab and trametinib treatment alone or in combination, 4 × 10^4^ of HT29, Pt-1, Pt-2 and Pt-3 cells were plated in triplicate in 24-well plate. 24 hours later cells were incubated with patritumab antibody at a final concentration of 10 μg/ml, trametinib at a final concentration of 5 nM, or a combination of both drugs. After 72 hours, cells were fixed in 4% formaldeyde for 15 minutes, then the cells were incubated with a solution of 0, 1% crystal violet in 10% ethanol. After 40 minutes the crystal violet solution was removed and cells washed with water and let to air-dry. Thereafter, crystals were solubilized in 10% acetic acid and absorbance was measured at 570 nm on a μQuant plate reader (BIO-TEK instruments inc, Milan, Italy). Each assay was repeated at least three times. The TUNEL assay was performed by In Situ Cell Death Detection Kit, Fluorescein (Roche, MI, Italy).

### Cell cycle analysis

After an overnight of serum starvation, HT29, Pt-1 and Pt-2 cell lines were incubated with 10 ng/ml HRG-β1 and 10 μg/ml patritumab antibody for the indicated times.

The cells were harvested by treatment with 0.25% trypsin, fixed with ice-cold 70% ethanol solution, and stained in PBS containing propidium iodide and RNase A overnight at 4°C. The DNA content of the cells was measured by fluorescence-activated cellsorting (FACS). Cell suspensions were analyzed on a FACSCalibur flow cytometer (Becton-Dickinson, San Josè, CA, USA) equipped with a 488 nm argon laser, after addition of 5 µl of a 1 mg/ml solution of propidium iodide to exclude non-viable cells. A total number of 1 × 10^4^ cells for each sample were acquired. The Cell-Quest software was used to analyze data.

### Tissue microarray construction

All colorectal cancers included in the study were histopathologically re-evaluated on haematoxylin and eosin stained slides and representative areas were marked prior to tissue microarray (TMA) construction.

Two core cylinders (1 mm diameter) were taken from CRC and deposited into two separate recipient paraffin blocks using a specific arraying device (Alphelys, Euroclone, Milan, Italy). In cases where informative results on TMA were absent due to missing tissue, no tumor tissue, we re-analyzed the correspondent routine tissue section. Two-mµ sections of the resulting microarray block were made and used for immunohistochemical (IHC) analysis after transferring them to SuperFrost Plus slides (Menzel-Gläser, Braunschweig, Germany).

### Immunohistochemistry

Immunohistochemical staining on TMA was performed using the following primary antibodies: anti-HER3 rabbit monoclonal antibody (D22C5) (Cell Signaling, Sial, Rome, Italy) and phospho-HER3 rabbit monoclonal antibody (Tyr1289-21D3) (Cell Signaling) in an automated immunostainer (Bond-III, Leica, Italy). A pH 8 buffer was used for the two antibodies as antigen retrieval according to the manufacturer’s protocol. Images were obtained at 20x magnification by using a light microscope (DM2000 LED, Leica) equipped with software able to capture images (Leica).

### Statistical methods

Levels of HER3 and pHER3 expression were scored semi-quantitatively based on staining intensity and distribution percentage using the immune-reactive score (IRS, staining intensity × percentage of positive cells). IRS was classified as low intensity and high intensity. The analysis by means of maximally selected log-rank statistics were performed to find optimal cut-offs for IRS capable of splitting patients into groups with different outcomes probabilities. The association between variables was tested by the Pearson Chi-Square test or Fisher’s Exact test, when appropriate. Hazard ratios (HR) and 95% confidence intervals (95% CI) were estimated for each variable using the Cox univariate model; a multivariate Cox proportional hazard model was developed using stepwise regression (forward selection, enter/remove limits *p* = 0.10 and *p* = 0.15 respectively), in order to identify independent predictors of outcomes; potential interactions between significant variables were taken into account when developing the multivariate model. Survival curves were calculated by the Kaplan-Meier product limit method from the date of the surgery until relapse/progression or death. The log-rank and Tarone-Ware tests were used to assess differences between subgroups. Significance was defined at the *p* ≤ 0.05 level.

Student *T* test was used to analyze cell lines proliferation, death data and cell cycle expressed as mean percentage. Student *T* test was also used to analyze the densitometry. Differences were considered statistically significant when *P* ≤ 0.05 (^*^P < 0.05, ^**^P < 0.001, ^***^P < 0.0001). All analyses were performed using SPSS^®^ (21.0), R^®^ (2.6.1) statistical programs (Chicago, Illinois).

## SUPPLEMENTARY MATERIALS FIGURES AND TABLE


