# Supplementary material for: Dual targeting of HER3 and MEK may overcome HER3-dependent drug-resistance of colon cancers
Source: Oncotarget. 2016 Aug 19;8(65):108463–79. doi: 10.18632/oncotarget.11400 (PMC5752456; doi:10.18632/oncotarget.11400)
Supplement: Supplementary file 1 [file oncotarget-08-108463-s001.pdf]

Dual targeting of HER3 and MEK may overcome HER3-dependent drug-resistance of colon cancers

Supplementary Materials

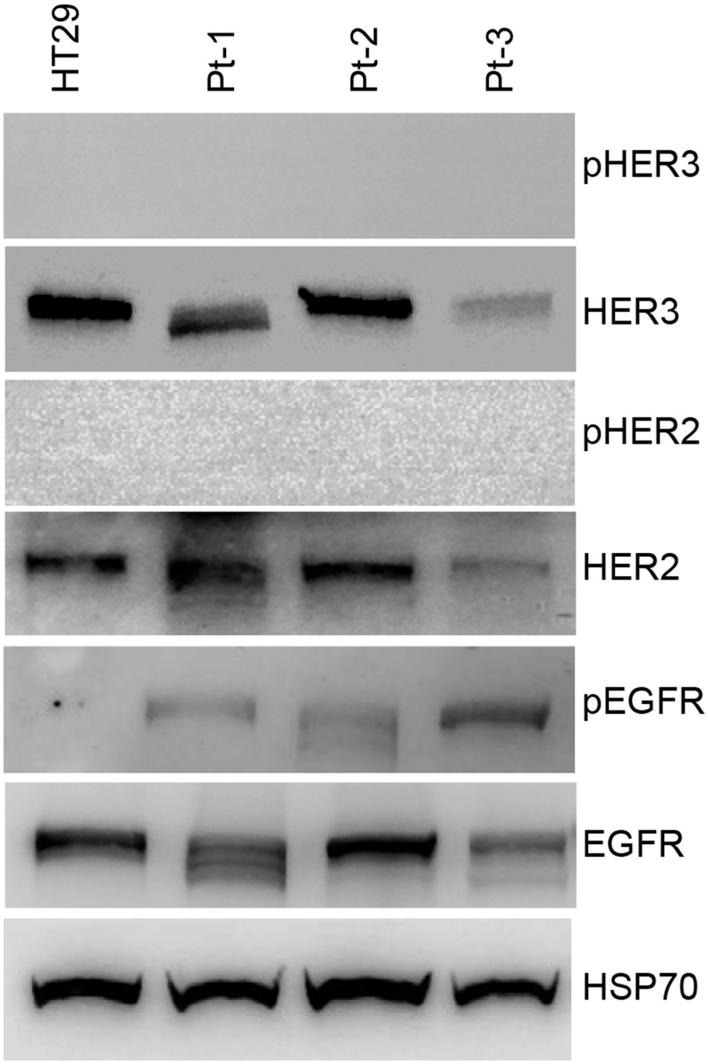

Supplementary Figure S1: Expression levels of EGFR family members in HT29 cells and in patient-derived colon cancer cell lines. HT29, Pt-1, Pt-2, and Pt-3 cell lines were analysed for the expression, at the basal level, of total and phospho-HER3, HER2, and EGFR. Total cell extracts were separated by SDS-page and immunoblotted with the indicated antibodies. Anti-HSP70 was used to validate equivalent amounts of loaded proteins in each lane.

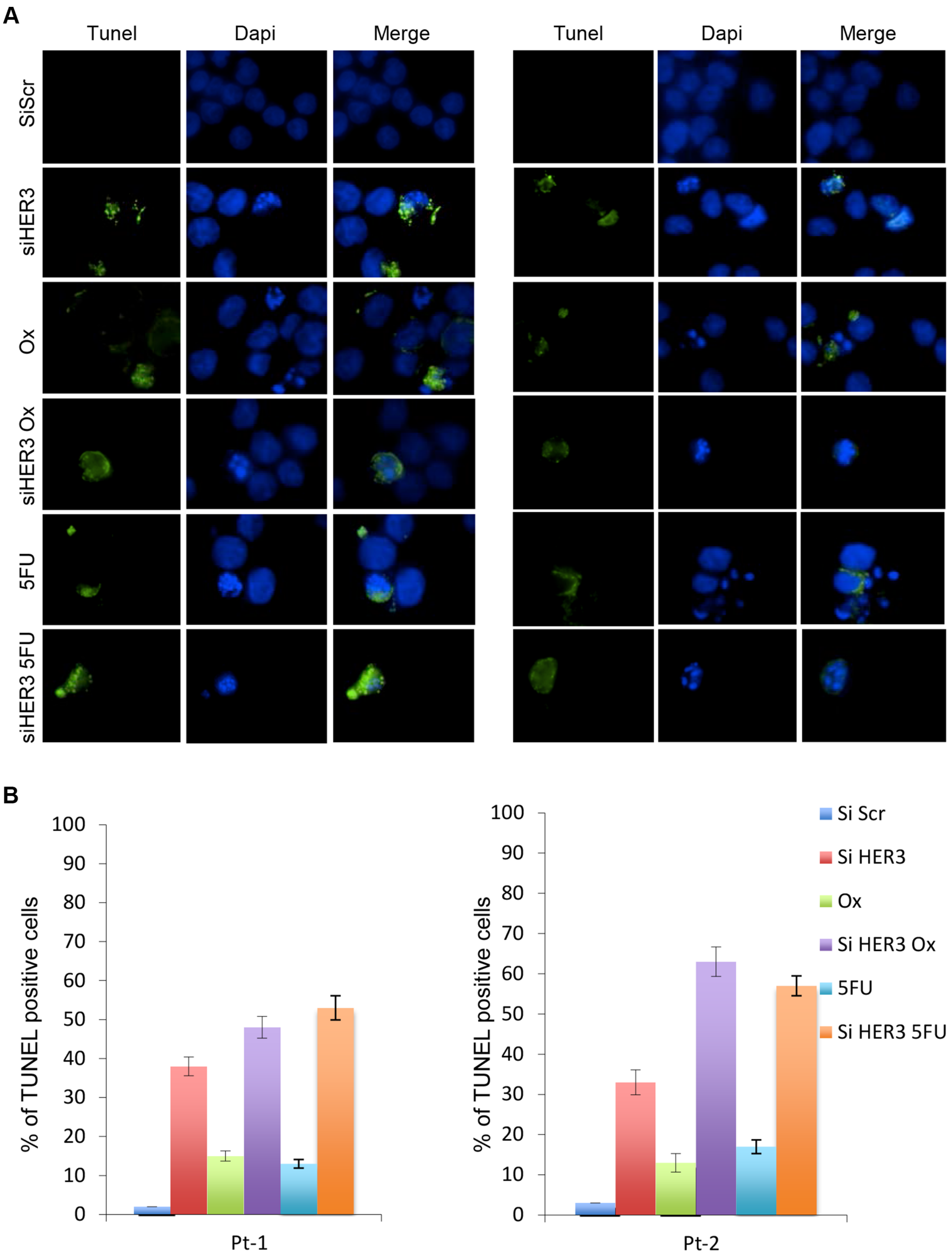

Supplementary Figure S2: TUNEL assay demonstrates that interference with HER3 expression induces apoptosis of Pt-1 and Pt-2 cells and sensitizes the cells to chemotherapy. Cell death of transiently transfected siscr and siHER3 Pt-1 (**A**, upper left panels), and Pt-2 cells (A, upper right panels), treated or not with 5-FU and Ox for 48 hours, was evaluated by TUNEL assay. The percentage of TUNEL positive cells is presented as mean +/– standard deviation of three independent experiments (**B**).

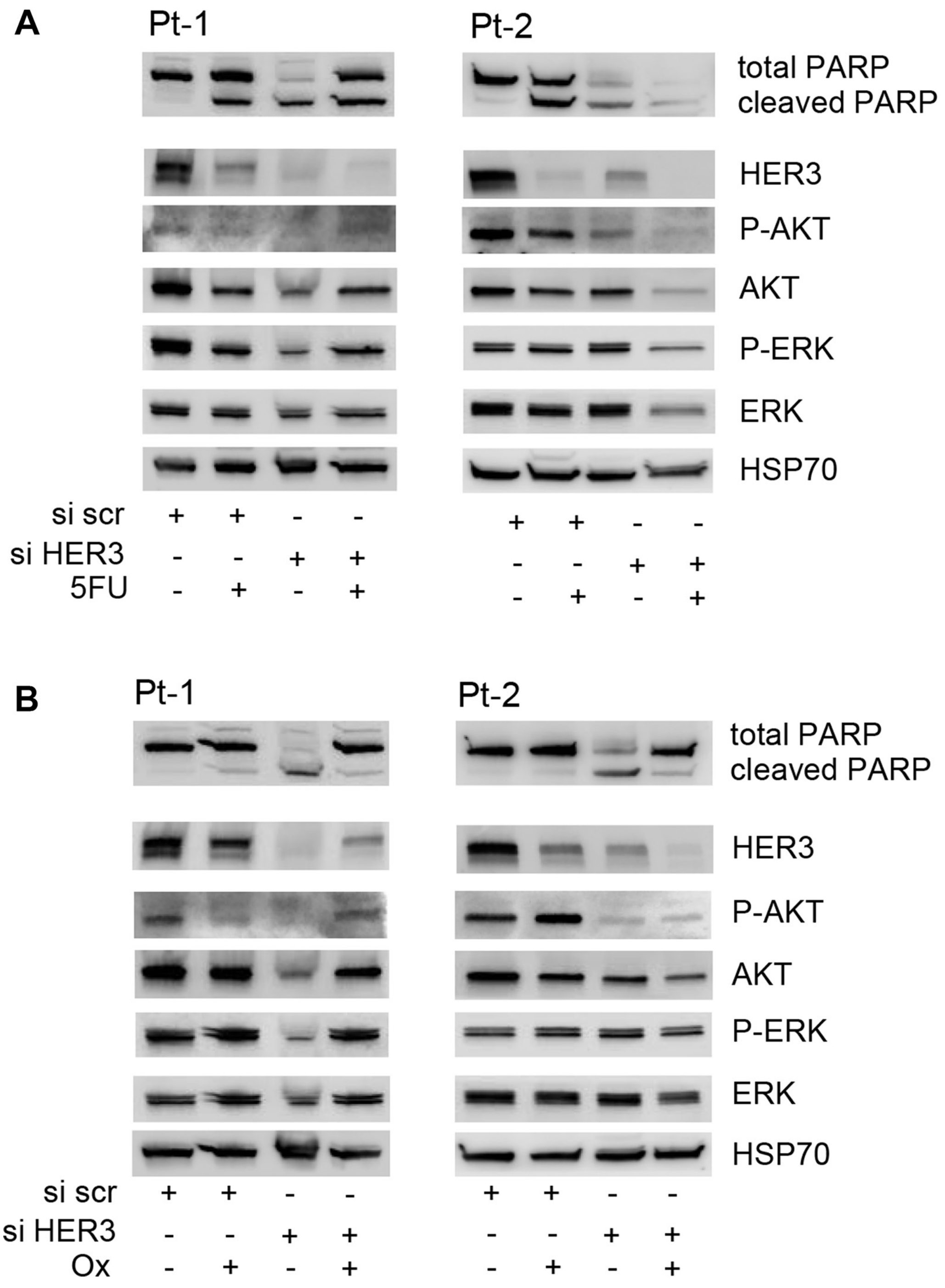

Supplementary Figure S3: Depletion of HER3 expression by a specific second siRNA induces apoptosis and sensitizes colon cancer cells to 5-FluoroUracil and Oxaliplatin. Total cell lysates from transiently transfected si scr and siHER3 Pt-1 and Pt-2 cells, treated or not with 5-FU (**A**) and Ox (**B**) were analyzed by immunoblot to evaluate the expression of HER3, total and p-AKT, total and p-ERK, and PARP. The anti-HSP70 antibody was used to validate equivalent amount of loaded proteins in each lane.

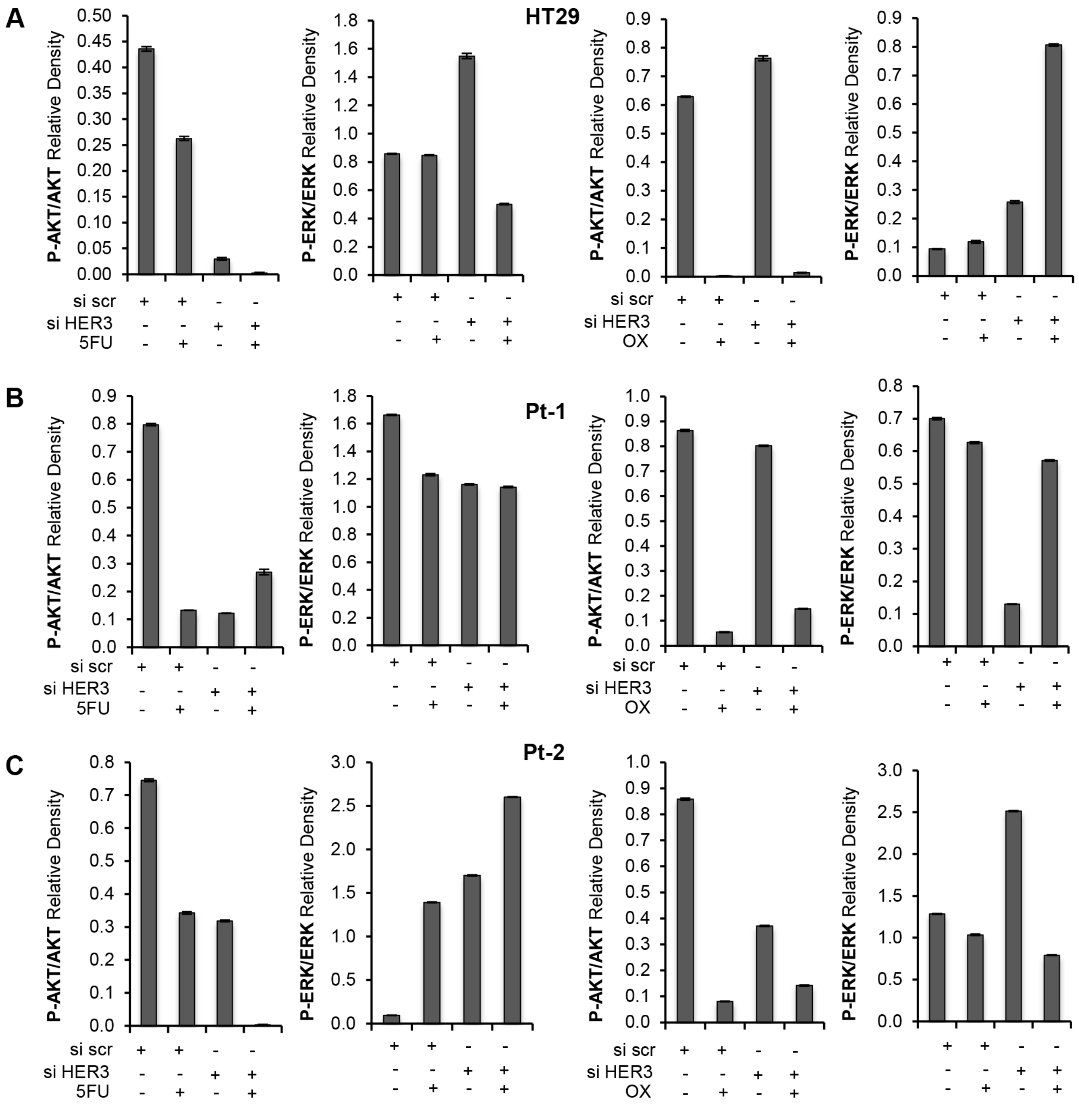

Supplementary Figure S4: Densitometry analysis of P-AKT and P-ERK of patient-derived cells, upon siHER3 and/or 5FU and Ox treatments. Densitometry analysis of P-AKT/AKT and P-ERK/ERK of HT29 (**A**), Pt-1 (**B**), and Pt-2 (**C**), transiently transfected with siscr and siHER3 treated or not with 5-FU (A, B, and C, left panels) and Ox (A, B, and C, right panels) for 48 hours.

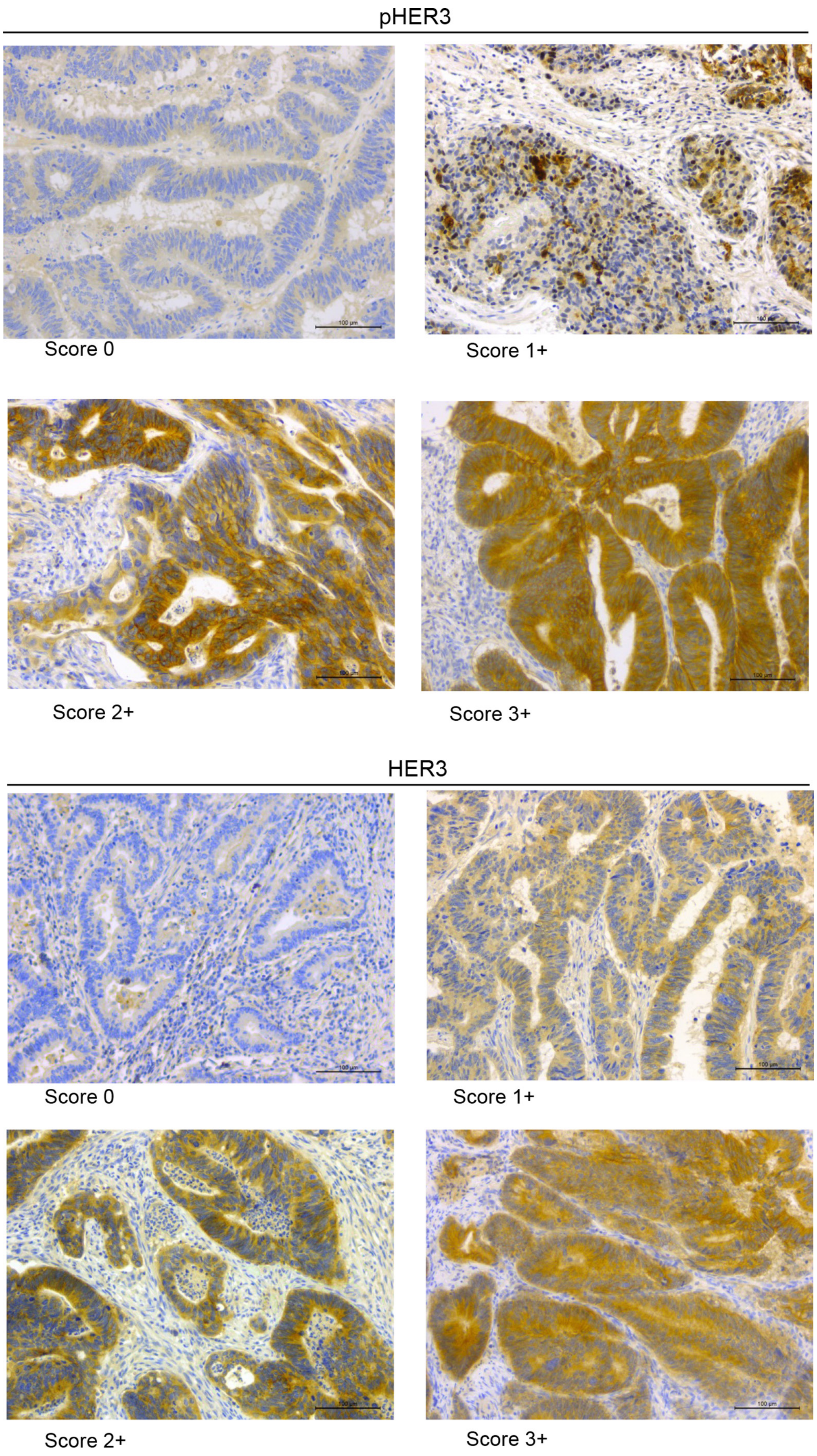

Supplementary Figure S5: Phospho-HER3 and total HER3 expression score criteria. Four representative CRC cases stained for phospho-HER3 (**A**) and total HER3 (**B**) based on staining intensity. The cases represent score 0, 1+, 2+, and 3+ as indicated in the figure.

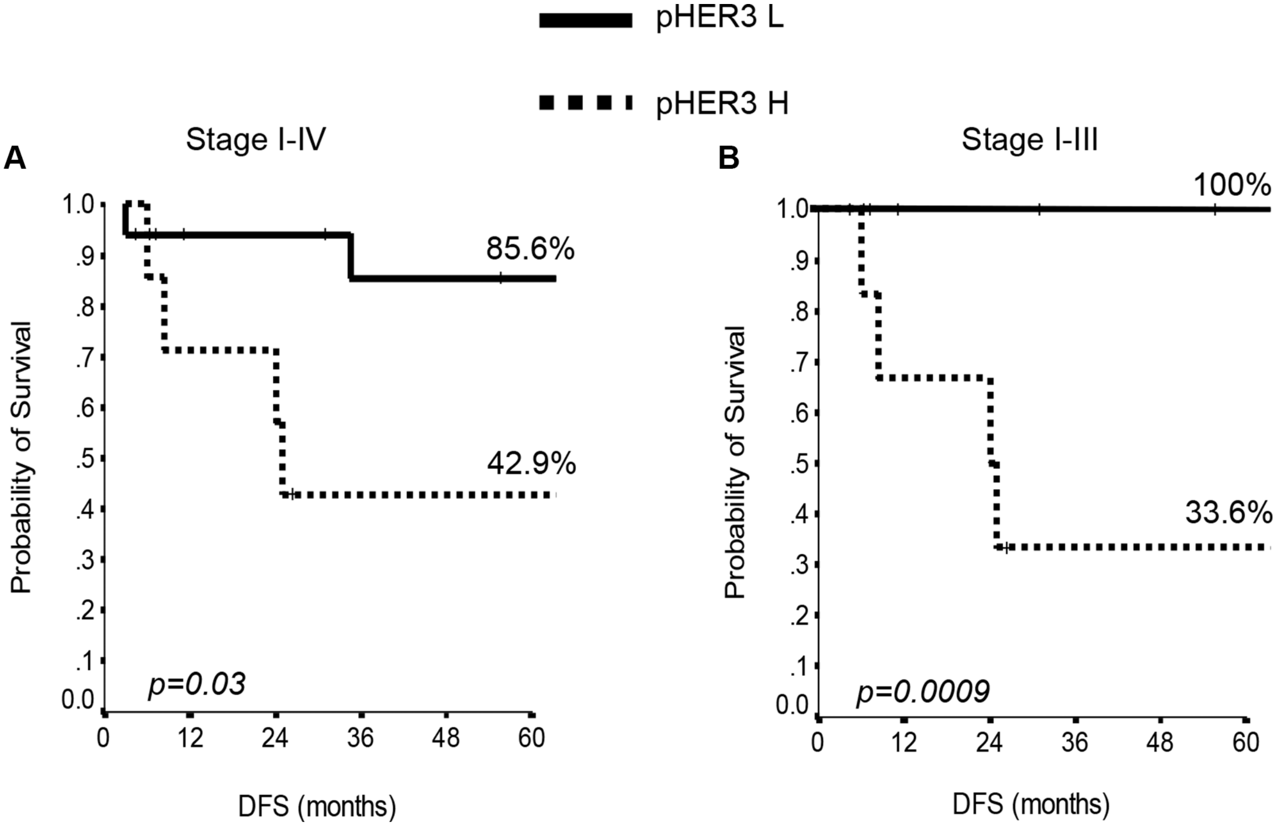

Supplementary Figure S6: p-HER3 correlation with disease-free survival is highly significant in low HER3-expressing CRC specimens. Kaplan-Meier estimates of DFS for pHER3 status in the subset of: (**A**) low HER3 expressing patient group (24 patients) in stage I–IV and (**B**) in stage I–III (21 patients) CRC patients.

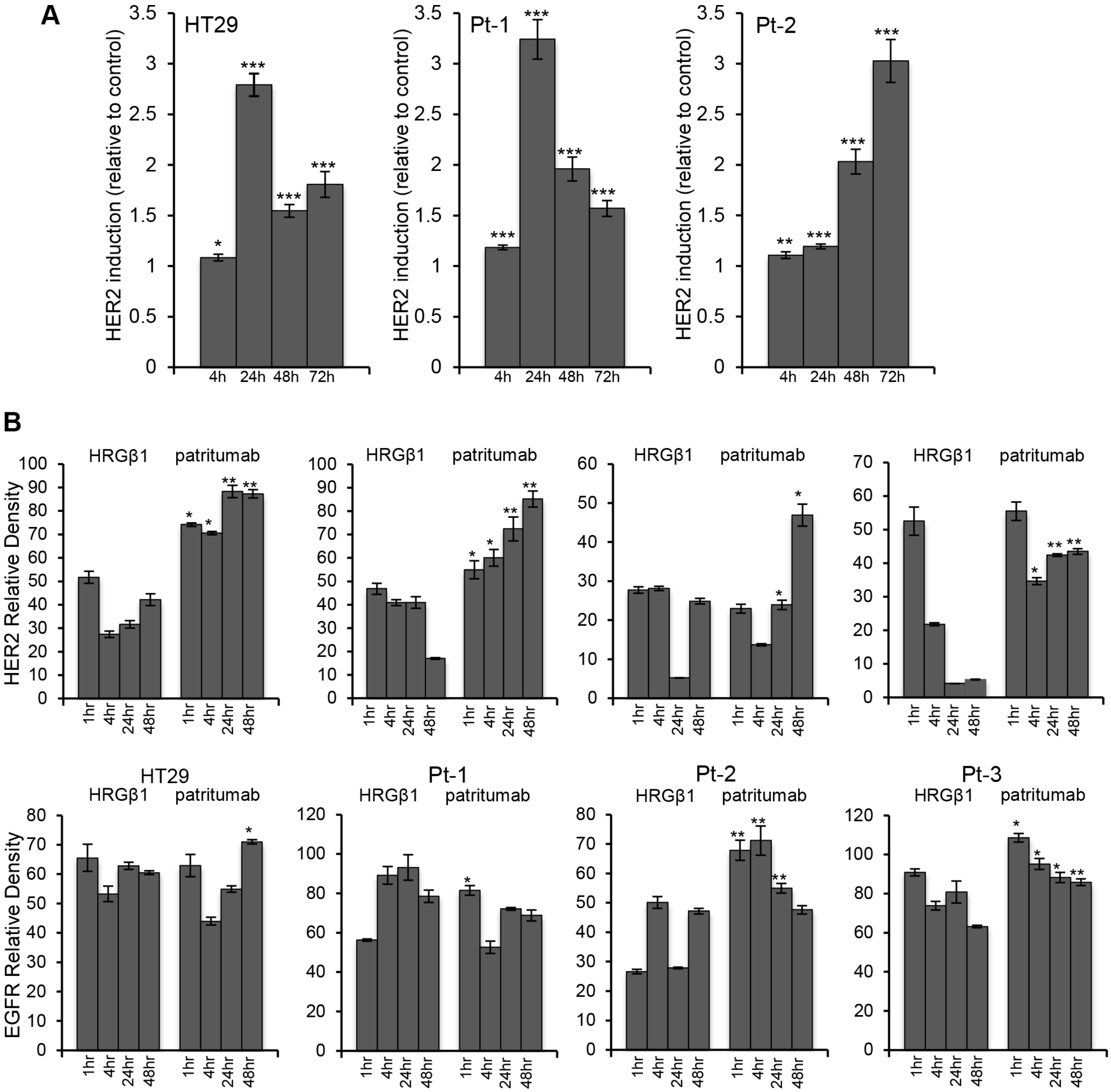

Supplementary Figure S7: Patritumab administration results in the up-regulation of HER2 and EGFR. (**A**) Fluorescence-activated cell sorting analysis of endogenous HER2 protein was done by a flow-cytometer after addition of 5 l of a 1 mg/ml solution of propidium iodide to exclude non-viable cells in HT29, Pt-1, and Pt-2, treated with 10 µg/ml patritumab and/or 10 ng/ml of HRG-β1 for the indicated times. At least 1×10^4 cells per sample were analysed. The results are presented as HER2 induction in patritumab-treated cells compared to HRG-β1-treated control cells. B. Densitometric analysis of HER2 and EGFR total proteins obtained 1, 4, 24, and 48 hours after treatment with 10 µg/ml patritumab and/or 10 ng/ml HRG-β1. Results represent mean ± standard deviation. $^{*}p < 0.05$; $^{**}p < 0.001$; $^{***}p < 0.0001$ compared to control.

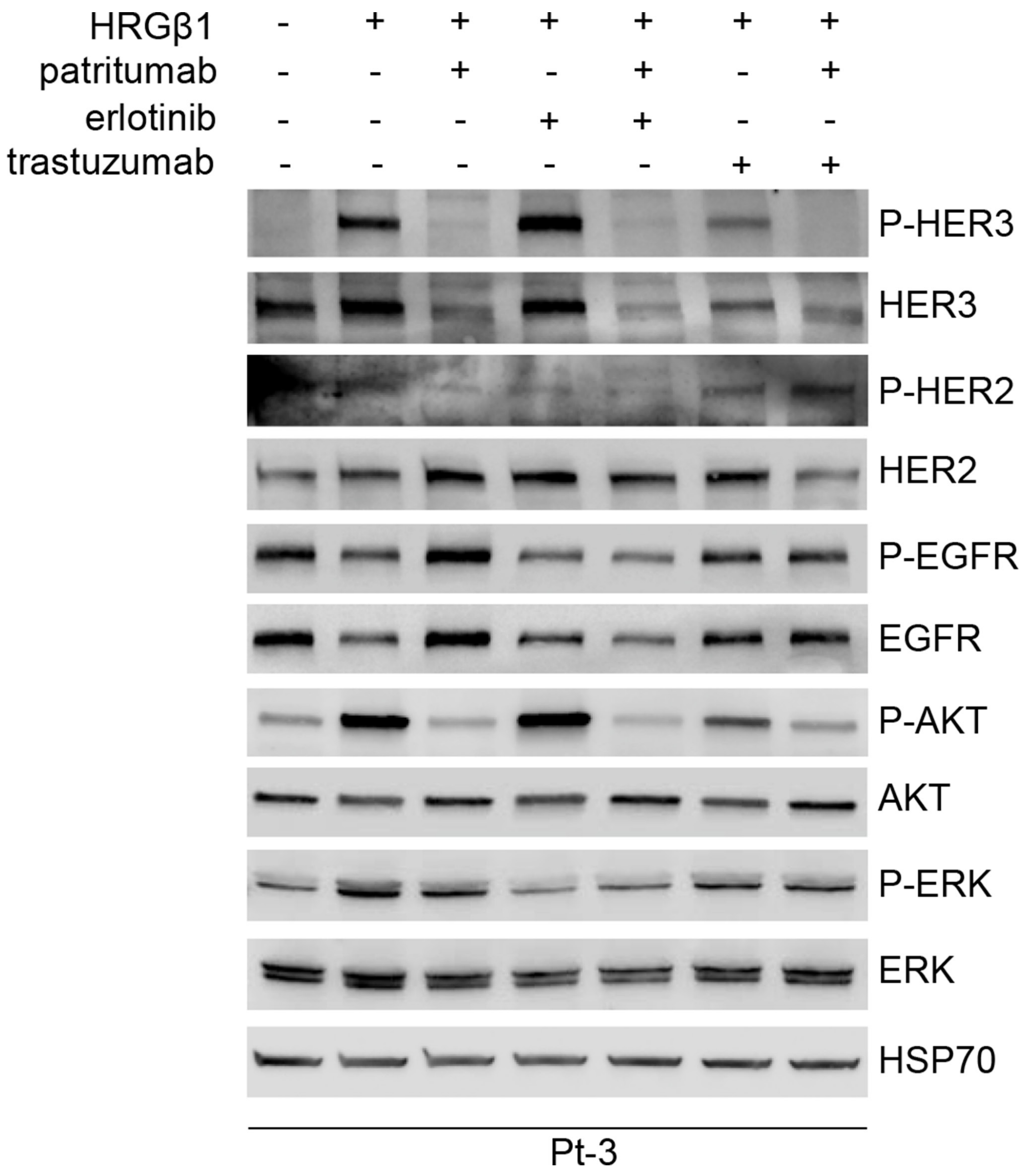

Supplementary Figure S8: The patritumab-induced activation of MAPK is reverted by HER2 and EGFR receptors signaling inhibition. Total cell lysates from Pt-3 cells incubated with 10 ng/ml HRG-β1, 10 μg/ml patritumab, 1 μM EGFR inhibitor erlotinib, and 25 μg/ml HER2 inhibitor trasuzumab for 4 hours, were analyzed by immunoblot to evaluate the expression of P-HER3, HER3, P-HER2, HER2, P-EGFR, EGFR, P-AKT, AKT, P-ERK, and ERK. The anti-HSP70 antibody was used to validate equivalent amount of loaded proteins in each lane.

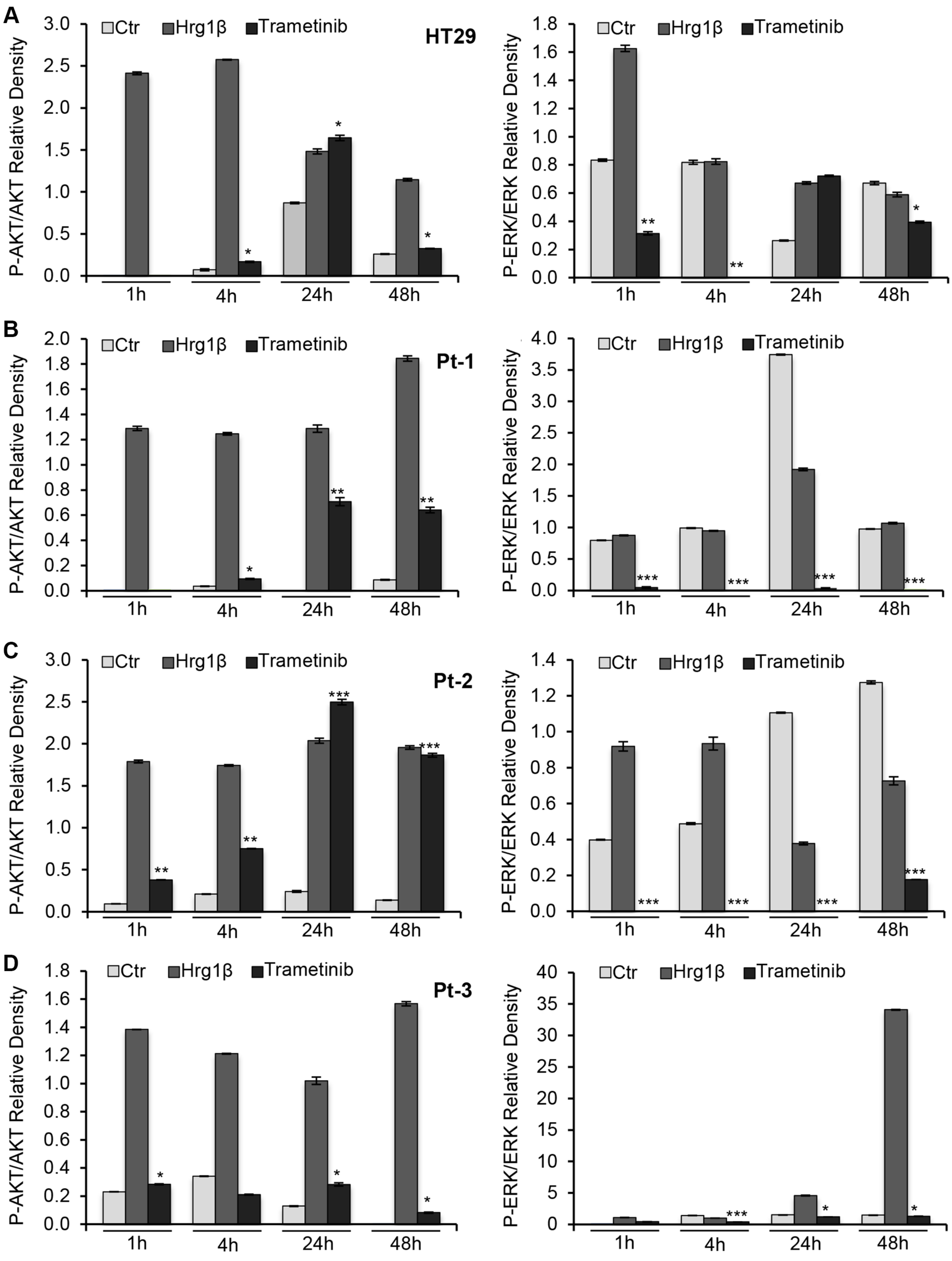

Supplementary Figure S9: Densitometric analysis of P-AKT and P-ERK upon trametinib treatment. Densitometric analysis of the proteins obtained 1, 4, 24, and 48 hours after treatment with 10 ng/ml HRG-β1 or 5 nM trametinib of HT29 (**A**), Pt-1 (**B**), Pt-2 (**C**), and Pt-3 (**D**) cells. Results represent mean ± standard deviation. $^{*}p < 0.05$; $^{**}p < 0.001$; $^{***}p < 0.0001$ compared to control.

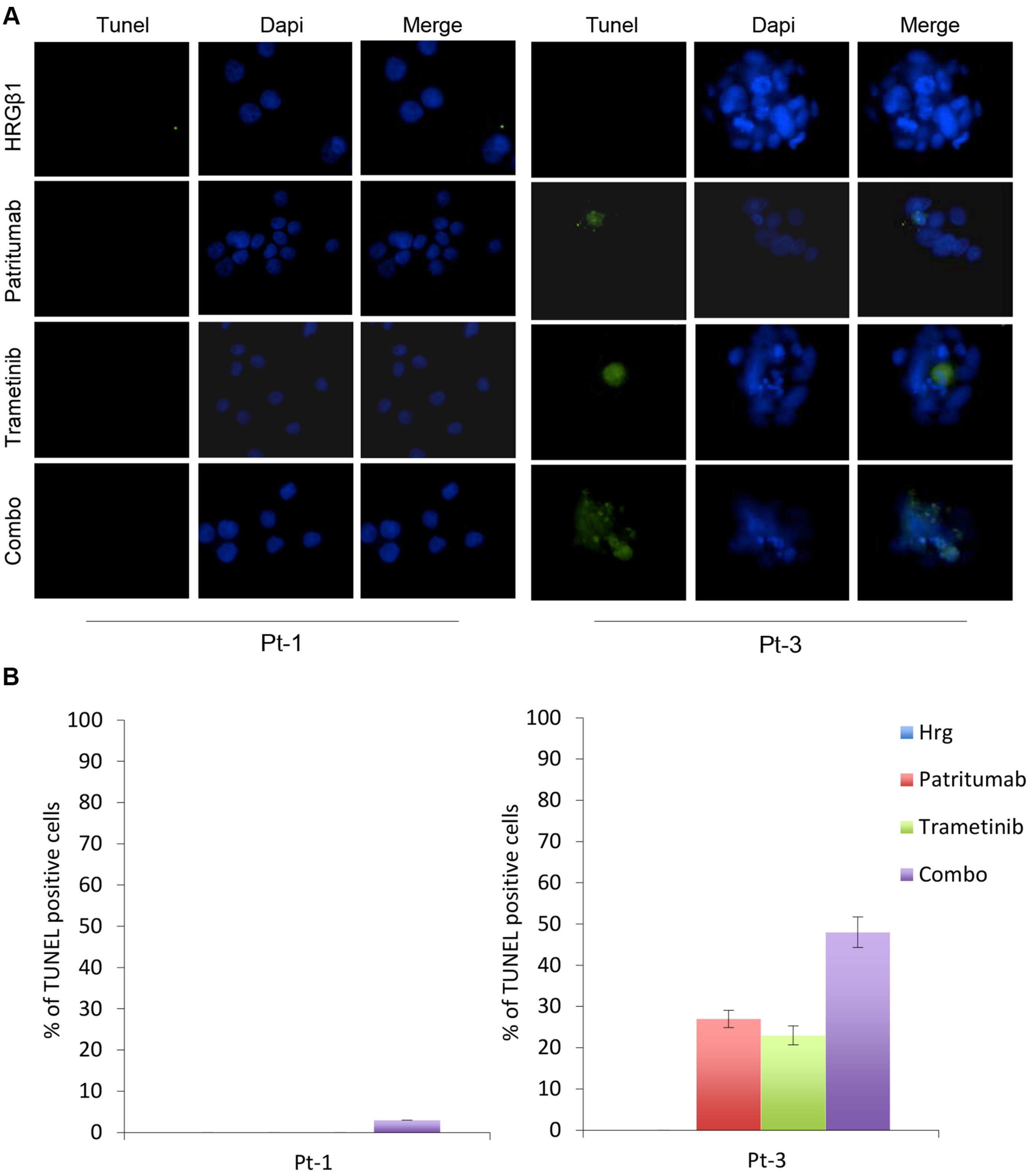

Supplementary Figure S10: TUNEL assay of Pt-1 and Pt-3 cells upon treatment with patritumab, trametinib, and the combination of both. Cell death of Pt-1, and Pt-3 cells upon treatment (**A**), was evaluated by TUNEL assay. The percentage of TUNEL positive cells is presented as mean +/–standard deviation of three independent experiments (**B**).

Supplementary Table S1: Oncogene status in patient-derived colon cancer cell lines

Cell line	KRAS	BRAF	PIK3CA	TP53
HT29	wt	V600E	P449T	R273H
Pt-1	wt	V600E	P449T	R273H
Pt-2	wt	wt	H1047R	R158A
Pt-3	G13	wt	E542K	wt

HT29 cells, and Pt-1, Pt-2, and Pt-3 patient-derived cell lines were analysed for the expression of KRAS, BRAF, PIK3CA, and p53 mutations.
